# Comment on: Post‑resuscitation diastolic blood pressure is a prognostic factor for outcomes of cardiac arrest patients

**DOI:** 10.1186/s40560-022-00646-z

**Published:** 2022-12-08

**Authors:** Romain Jouffroy, Benoît Vivien

**Affiliations:** 1 Service de Médecine Intensive Réanimation, Hôpital Universitaire Ambroise Paré, Assistance Publique-Hôpitaux de Paris, and Paris Saclay University, Gif-sur-Yvette, France; 2 SAMU de Paris, Service d’Anesthésie Réanimation, Hôpital Universitaire Necker-Enfants Malades, Assistance Publique-Hôpitaux de Paris, and Université Paris Cité, Paris, France

## To the editor,

We read with great interest the recent article published in the Journal by Chi et al. [1] reporting that diastolic blood pressure (DBP) after cardiac arrest (CA) resuscitation can predict outcomes, as a higher DBP level correlated with better prognosis after cardiogenic CA.

Whereas the authors must be congratulated for this interesting study, we believe that their results’ interpretation needs caution.

From a statistical point of view, either Area Under the Curve Receiver Operating (AUC-ROC) Characteristics analysis nor Generalized Additive Model (GAM) is statistical methods for prognostication. AUC-ROC aims to define the optimal cutoff between two groups. GAM is a statistical method for association, but not for causal association. Age-adjusted odd ratio had allow the reader to consider the association intensity with study endpoints. It is surprising to not consider in the multivariate analysis no flow duration, which is one Utstein variable [2]. Moreover, the better outcome among patients with higher DBP may per se partly be explained by a greater representation of patients with initial shockable rhythm in this subgroup [3], and it would have been interesting to assess the potential interaction between these two variables.

From a pathophysiological point of view, the higher DBP among survivors may partly be explained by a lower epinephrine administration requirement, e.g., reflecting a lower no flow duration and consequently a lower impact of the post-resuscitation syndrome [4] and/or of a targeted temperature of 33 °C inducing vasoconstriction, both not included in the multivariate analysis. Acidosis occurring after cardiac arrest is consequently lower in this subgroup, partly explaining lower negative effects on the vascular alpha-1-sympathomimetic receptor response to endogenous and exogenous sympathomimetic agents [5, 6].

Beyond these limitations, we agree with Chi et al. [1] that DBP after cardiac arrest resuscitation reflects a lower severity of the post-resuscitation syndrome. Further studies are needed to clarify the causal link between the outcome and DBP after cardiac arrest.

## Data Availability

Not applicable.
